# Motion around triangular points in the restricted three-body problem with radiating heterogeneous primaries surrounded by a belt

**DOI:** 10.1038/s41598-020-75174-7

**Published:** 2020-11-02

**Authors:** Jagadish Singh, Sunusi Haruna

**Affiliations:** 1 Department of Mathematics, Faculty of Physical Sciences, Ahmadu Bello University, Zaria, Nigeria; 2 Basic Studies Department, School of General Studies, Kano State Polytechnic, Kano, Nigeria

**Keywords:** Astronomy and astrophysics, Mathematics and computing

## Abstract

The present paper studies the locations and linear stability of the triangular equilibrium points when both primaries are radiating and considered as heterogeneous spheroid with three layers of different densities. Additionally, we include the effects of small perturbations in the Coriolis and centrifugal forces and potential from a belt (circumbinary disc). It is observed that the positions of the triangular equilibrium points are substantially affected by all parameters (except a perturbation in Coriolis force) involved in the system.The stabilty of motion is found only when $0 < \mu < \mu_{c}$, where $\mu_{c}$ is the critical mass value which depends on the combined effect of radiation pressures and heterogeneity of the primaries, small perturbations and the potential from a belt.It is also seen that the Coriolis force and the belt have stabilizing effect,while the centrifugal force, radiation and heterogeineity of the primaries have destabilizing behaviour.The net effect is that the size of the region of stability decreases when the value of these parameters increases where $\mu$ is the mass ratio and $k_{1} ,k_{2}$ characterize heterogeneity of both primaries. A practical application of this model could be the study of motion of a dust grain near the heterogeneous and luminous binary stars surrounded by a belt.Finally, we carried out and discuss numerical experiments aiming at computing the positions of triangular points and critical masses of three binary systems: Archid, Xi Booties and Kruger 60.

## Introduction

Recent advancements in the mathematical theory of differential equations and dynamical system have been aided by studies carried out in the three-body problem (3BP).The 3BP consists of, for a given set of initial conditions, the motion of three bodies governed by their mutual gravitational attraction. It has found wide-spread applications in the field of dynamical astronomy (Murray and Dermott^1^.

The restricted three-body problem (R3BP) is a modified form of the 3BP and differs from the 3BP in the sense that two of the bodies have finite masses and are often referred to as primaries while the third body whose mass is infinitesimally small is often called the infinitesimal body. Further, motion of the primaries is not influenced by the gravitational force exerted by the infinitesimal body where as their gravitational attraction completely determines the motion of the infinitesimal body. Hence the R3BP is to determine the motion of the infinitesimal body under the gravitational influence of the primaries. In the instance whereby the primaries move in circular orbits about their common centre of mass, such set up is referred to the circular restricted three-body problem (CR3BP). The CR3BP is still not solvable in closed form. However, some particular solutions exist allowing an insight into the problem. To first order (linearization), Lagrange^2^ showed the existence of five equilibrium solutions or libration points of the CR3BP. These consist of three collinear and two triangular points which are denoted by $L_{i} (i = 1,2,3)$ and $L_{4}$, $L_{5}$ respectively.

The velocity and acceleration of the infinitesimal body when it is placed at any of these equilibrium points are equal to zero. A useful property of the CR3BP which is the possible position of equilibrium occurs when the three bodies form an equilateral triangle which can be observed in the motion of Trojan asteroid around the triangular points $L_{4}$ and L_5_. In this case, the Asteroids are influenced only by the gravitational forces of the Sun and Jupiter, and the orbit of Jupiter around the Sun is assumed a fixed ellipse. In the classical context all bodies are considered as Newtonian point-masses with no physical extension and so Coriolis and centrifugal forces, radiation pressure, gravitational potential from the belt, Poynting-Robertson drag etc. do not act on them(Singh and Amuda^3^). In the Solar and Stellar systems, a vast number of planets, their satellites and stars are sufficiently oblate or triaxial in shapewith layers of different densities (heterogeneous). The lack of sphericity or heterogeneity of a body causes large perturbations (Singh and Richard^4^).

It is known that the classical CR3BP did not discuss the motion of the infinitesimal body when one or both primaries is a source of radiation.Radzievsky^5^ was first to discuss this problem by formulating thephoto-gravitational CR3BP which he applied to the Sun-planet-particle and Galaxy Kernel. The study of stability of the equilibrium points was examined by Szebehely^6^. He established in the linear sense that the collinear points are in general unstable while the triangular points are stable for $0 < \mu < \mu_{c} = 0.3852...$, Simmons et al.^7^, on the other hand discussed the complete solution and linear stability of the equilibrium points for all the values of radiation pressure of both luminous bodies. They observed that when both large masses are luminous, the inner $L_{1}$ point can be stable. Subsequently, the effect of oblateness and radiation pressure of the primaries on location and linear stability of the triangular points was investigate by Singh and Ishwar^8^ and found that the points are stable for $0 < \mu < \mu_{c} = 0.3852...$ and unstable for $\mu_{c} < \mu < \frac{1}{2}$ with $\mu_{c}$ characterized by the radiation and oblateness coefficients. In furtherance, AbdulRaheem and Singh^9^ examined the combined effect of perturbations, radiation and oblateness on positions and stability of equilibrium points in the R3BP. The triangular points are stable and unstable under same condition as in Singh and Ishwar^8^. They observed further that Coriolis force has a stabilizing effect, while the centrifugal force, radiation and oblateness of the primaries have destabilizing effects. The presence of any destabilizing force renders unstable the stability of the stabilizing agents, and consequently decreases the range of stability of the triangular points.

The R3BP in the presence of a disc was studied by Yeh and Jiang^10^. In this study, they investigated the possible chaotic and regular orbits for the disc-star-planet system and found that the influence from the disc can alter locations of equilibrium points and the stability pattern. Singh and Taura^11^ however investigated the motion in a generalized R3BP when both primaries are radiating and oblate together while also considering the effect of a gravitational potential caused by a massive belt.The stability analysis of the equilibrium points shows that collinear points remain unstable, while the triangular points are conditionally stable. Subsequently Suraj et al.^12^, discussed the equilibrium solutions of the planar R3BP when both primaries are heterogeneous oblate spheroids with three layers of different densities and sources of radiation. They observed that the collinear points are unstable, while the triangular points are stable for the mass parameter $0 < \mu < \mu_{C}$_._

In this study, we intend to modify the critical mass value $\mu_{c}$ of Suraj et al.^12^, we also wish to extend their work by including the effects of small perturbations in the Coriolis and centrifugal forces and potential from a belt (circumbinary disc).The paper is organized as follows: Equations of motion presents equations of motion, Location of triangular equilibrium points locates the triangular points. Linear stability of triangular points examines their stability. Lastly the discussion and conclusion are provided in Discussion and Conclusion, respectively.

**Figure 1:**
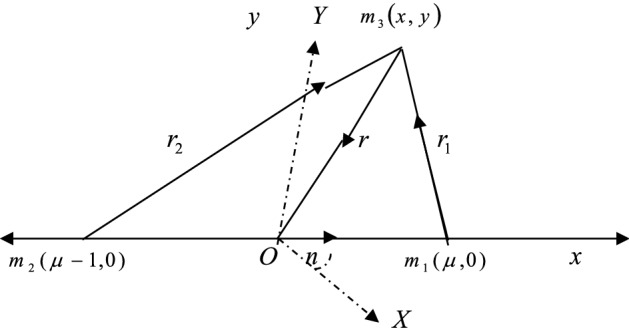
Configuration of the CR3BP.

Figure 1 illustrates the configuration of the CR3BP. Here we draw the case for two equal-mass primary with O being the center of mass.

## Equations of motion

Let $m_{1} ,m_{2}$, and $m_{3}$ be the masses of the primary, secondary, and the infinitesimal body (third body ) respectively. The third body is moving under the influence of both primaries which are heterogeneous with three layers, sources of radiation and surrounded by a belt. We take a coordinate system $O(x,y,z)$ with the origin at the centre of mass of the primaries and the x-axis is the line joining them, while the *y*-axis is perpendicular to it, and the *z*-axis is perpendicular to the orbital plane of the primaries. Let the coordinate system rotate with angular velocity n about the *z*-axis. In the aforesaid coordinate system and dimensionless variables, the equations of motion of the third body in the frame work of the CR3BP when both the primaries are heterogeneous, radiating and surrounded by a belt under the influence of small perturbations in the Coriolis and centrifugal forces, can be written, following the work of Suraj et al.^12^ and Singh and Taura^11^, as

$$\begin{gathered} \ddot{x} - 2n\alpha \dot{y} = \Omega_{x} \hfill \\ \ddot{y} + 2n\alpha \dot{x} = \Omega_{y} \hfill \\ \end{gathered}$$

with

1 $$\Omega = \frac{{n^{2} \beta }}{2}\left( {x^{2} + y^{2} } \right) + q_{1} \left( {\frac{1 - \mu }{{r_{1} }} + \frac{{k_{1} }}{{2r_{1}^{3} }}} \right) + q_{2} \left( {\frac{\mu }{{r_{2} }} + \frac{{k_{2} }}{{2r_{2}^{3} }}} \right) + \frac{{M_{b} }}{{\left( {r^{2} + T^{2} } \right)^{\frac{1}{2}} }}$$

$$\begin{aligned} & 0 < \mu = \frac{{m_{2} }}{{m_{1} + m_{2} }} \le \frac{1}{2},m_{1} > m_{2} > > m_{3} \\ & r_{1}^{2} = \left( {x - \mu } \right)^{2} + y^{2} ,\quad r_{2}^{2} = \left( {x - \mu + 1} \right)^{2} + y^{2} ,\quad r^{2} = x^{2} + y^{2} , \\ & n^{2} = 1 + \frac{3}{2}k_{3} + \frac{{2M_{b} r_{c} }}{{\left( {r_{c}^{2} + T^{2} } \right)^{\frac{3}{2}} }} \\ \end{aligned}$$

$k_{i} (i = 1,2,3)$ terms appear on account of both primaries being heterogeneous spheroid with three layers of different densities (Suraj et al.^12^).

$\mu$ is the mass parameter, $n$ is the mean motion of the primaries; $r_{1}$ and $r_{2}$ are the distances of the third body from the primaries. $q_{i} (i = 1,2),$ are the radiation factors of the primary and the secondary, respectively and given by $F_{Pi} = F_{gi} (1 - q_{i} )$ such that $0 < (1 - q_{i} ) < < 1$ (Radzievsky^5^), where $F_{gi}$, $F_{Pi}$_7_ are respectively the gravitational and radiation pressure forces. $\alpha = 1 + \varepsilon ,\varepsilon < < 1$, $\beta = 1 + \varepsilon^{\prime}$, $\varepsilon^{\prime} < < 1$ are the parameters for the Coriolis and centrifugal forces, respectively to which small perturbations $\varepsilon$ and $\varepsilon^{\prime }$ are given. $M_{b}$ is the total mass of the belt(circular cluster of material points), $r$ is the radial distance of the infinitesimal body, while $r_{c}$ is that in the classical case. $T = a + b$; $a$ and $b$ are parameters which determine the density profile of the belt. The parameter $a$ controls the flatness of the profile and is known as flatness parameter, while $b$ controls the size of the core of the density profile and is called the core parameter.

## Location of triangular equilibrium points

The equilibrium points represent stationary solutions of the CR3BP. These points are the singularities of the manifold of the components of the velocity and the coordinates. They are found by setting $\dot{x} = 0 = \dot{y} = \ddot{x} = \ddot{y}$ in the Eq. (1) of motion, i.e., they are solutions of equations.

$$\Omega_{x} = n^{2} \beta x - \frac{{\left( {1 - \mu } \right)\left( {x - \mu } \right)q_{1} }}{{r_{1}^{3} }} - \frac{{3\left( {x - \mu } \right)k_{1} q_{1} }}{{2r_{1}^{5} }} - \frac{{\mu \left( {x - \mu + 1} \right)q_{2} }}{{r_{2}^{3} }} - \frac{{3\left( {x - \mu + 1} \right)k_{2} q_{2} }}{{2r_{2}^{5} }} - \frac{{M_{b} x}}{{\left( {r^{2} + T^{2} } \right)^{\frac{3}{2}} }} = 0$$ and

3 $$\Omega_{y} = n^{2} \beta y - \frac{{\left( {1 - \mu } \right)yq_{1} }}{{r_{1}^{3} }} - \frac{{3k_{1} q_{1} y}}{{2r_{1}^{5} }} - \frac{{\mu yq_{2} }}{{r_{2}^{3} }} - \frac{{3k_{2} q_{2} y}}{{2r_{2}^{5} }} - \frac{{M_{b} y}}{{\left( {r^{2} + T^{2} } \right)^{\frac{3}{2}} }} = 0$$

Re-writing these equations, we have.

4 $$\begin{aligned} & x\left[ {n^{2} \beta - \frac{{q_{1} \left( {1 - \mu } \right)}}{{r_{1}^{3} }} - \frac{{3k_{1} q_{1} }}{{2r_{1}^{5} }} - \frac{{\mu q_{2} }}{{r_{2}^{3} }} - \frac{{3k_{2} q_{2} }}{{2r_{2}^{5} }} - \frac{{M_{b} }}{{\left( {r^{2} + T^{2} } \right)^{\frac{3}{2}} }}} \right] + \frac{{q_{1} \mu \left( {1 - \mu } \right)}}{{r_{1}^{3} }} + \frac{{3\mu k_{1} q_{1} }}{{2r_{1}^{5} }} + \frac{{\mu^{2} q_{2} }}{{r_{2}^{3} }} - \frac{{\mu q_{2} }}{{r_{2}^{3} }} + \frac{{3\mu k_{2} q_{2} }}{{2r_{2}^{5} }} \\ & \quad - \frac{{3k_{2} q_{2} }}{{2r_{2}^{5} }} = 0 \\ \end{aligned}$$

and

5 $$y\left[ {n^{2} \beta - \frac{{q_{1} \left( {1 - \mu } \right)}}{{r_{1}^{3} }} - \frac{{3k_{1} q_{1} }}{{2r_{1}^{5} }} - \frac{{\mu q_{2} }}{{r_{2}^{3} }} - \frac{{3k_{2} q_{2} }}{{2r_{2}^{5} }} - \frac{{M_{b} }}{{\left( {r^{2} + T^{2} } \right)^{\frac{3}{2}} }}} \right] = 0$$

The triangular points are the solutions of Eqs. (4) and (5) when $y \ne 0$.

Solving, we have

6 $$\left( {1 - \mu } \right)\left[ {n^{2} \beta - \frac{{q_{1} }}{{r_{1}^{3} }} - \frac{{M_{b} }}{{\left( {r^{2} + T^{2} } \right)^{\frac{3}{2}} }}} \right] = \frac{{3k_{1} q_{1} }}{{2r_{1}^{5} }}$$

and

7 $$\mu \left[ {n^{2} \beta - \frac{{q_{2} }}{{r_{2}^{3} }} - \frac{{M_{b} }}{{\left( {r^{2} + T^{2} } \right)^{\frac{3}{2}} }}} \right] = \frac{{3k_{2} q_{2} }}{{2r_{2}^{5} }}$$

Now, if the primaries are neither heterogeneous nor radiating and there is no potential from a belt, then $n = 1$, $q_{i} \left( {i = 1,2} \right) = 1$, $M_{b} = 0$ and $k_{i} \left( {i = 1,2,3} \right) = 0$. Consequently, Eqs. (6) and (7) reduce to

8 $$r_{1}^{3} = \frac{1}{\beta }\quad {\text{and}}\quad r_{2}^{3} = \frac{1}{\beta }$$

Now, we assume that the primaries are luminous and heterogeneous and there is potential from a belt, then the $r_{1}$ and $r_{2}$ will take the form,

9 $$r_{1} = \frac{1}{{\beta^{\frac{1}{3}} }} + \varepsilon_{1} {\text{ and }}r_{2} = \frac{1}{{\beta^{\frac{1}{3}} }} + \varepsilon_{2}$$

where $$\left| {\varepsilon_{i} } \right| < < 1$$ , for i = 1,2.

Substituting the value of $n$, and $r_{i} (i = 1,2)$ from (2) and (9), in (6) and (7) respectively and putting $\beta = 1 + \varepsilon^{\prime}$, $q_{i} = 1 - p_{i}$, $\left| {p_{i} } \right| < < 1$ while restricting to only linear terms in $\varepsilon_{i} ,k_{i} ,p_{i} ,\varepsilon^{\prime }$ and $M_{b}$, we obtain

10 $$\varepsilon_{1} = \frac{{k_{1} }}{{2\left( {1 - \mu } \right)}} - \frac{{k_{3} }}{2} - \frac{{p_{1} }}{3} - \frac{{M_{b} \left( {2r_{c} - 1} \right)}}{{3\left( {r_{c}^{2} + T^{2} } \right)^{\frac{3}{2}} }}$$

$$\varepsilon_{2} = \frac{{k_{2} }}{2\mu } - \frac{{k_{3} }}{2} - \frac{{p_{2} }}{3} - \frac{{M_{b} \left( {2r_{c} - 1} \right)}}{{3\left( {r_{c}^{2} + T^{2} } \right)^{\frac{3}{2}} }}$$

Substituting these values in Eq. (9), we have

11 $$\begin{aligned} & r_{1} = \frac{1}{{\beta^{\frac{1}{3}} }} + \frac{{k_{1} }}{{2\left( {1 - \mu } \right)}} - \frac{{k_{3} }}{2} - \frac{{p_{1} }}{3} - \frac{{M_{b} \left( {2r_{c} - 1} \right)}}{{3\left( {r_{c}^{2} + T^{2} } \right)^{\frac{3}{2}} }} \\ & r_{2} = \frac{1}{{\beta^{\frac{1}{3}} }} + \frac{{k_{2} }}{2\mu } - \frac{{k_{3} }}{2} - \frac{{p_{2} }}{3} - \frac{{M_{b} \left( {2r_{c} - 1} \right)}}{{3\left( {r_{c}^{2} + T^{2} } \right)^{\frac{3}{2}} }} \\ \end{aligned}$$

Substituting Eq. (11) in (2) i.e.,

$$r_{1}^{2} = \left( {x - \mu } \right)^{2} + y^{2}$$

$$r_{2}^{2} = \left( {x - \mu + 1} \right)^{2} + y^{2}$$

and solving them for $x$ and $y$_;_ we neglect products and higher order terms of small quantities. Consequently, we obtain the triangular equilibrium points $L_{4} (x,y)$ and $L_{5} (x, - y)$ as:

12 $$x = \mu - \frac{1}{2} + \frac{1}{2}\left( {\frac{{k_{2} }}{\mu } - \frac{{k_{1} }}{{\left( {1 - \mu } \right)}}} \right) - \left( {\frac{{p_{2} - p_{1} }}{3}} \right)$$

$$y = \pm \frac{\sqrt 3 }{2}\left[ {1 - \frac{4}{9}\varepsilon^{\prime} + \frac{{k_{1} }}{{3\left( {1 - \mu } \right)}} + \frac{{k_{2} }}{3\mu } - \frac{{2k_{3} }}{3} - \frac{{2p_{1} }}{9} - \frac{{2p_{2} }}{9} - \frac{{4M_{b} \left( {2r_{c} - 1} \right)}}{{9\left( {r_{c}^{2} + T^{2} } \right)^{\frac{3}{2}} }}} \right]$$

The values of $x$ and $y$ obtained in (12) are the coordinates of triangular equilibrium points, by virtue of the two triangles they form with the line joining the primaries and are denoted by $L_{4,5} (x, \pm y)$. It shows that the position of the triangular points are affected by a perturbation of the centrifugal force, the mass parameter ,radiation pressures and heterogeneity of the primaries and the potential from the belt.

Next, we wish to compute Eq. (12) numerically for the binary systems **Archid**, **Xi Bootis** and **Kruger 60.** For the masses of the primaries, their mass ratio and their radiation pressure factors, we adopt the numerical data presented by Singh and Umar^13^ in Table 1 as follows.

**Table 1:** Mass parameter and radiation pressure for binary systems **Archid**, **Xi Bootis** and **Kruger 60.**

Binary system	*M*_1_,* Mass*	*M*_2_,* Mass*	*µ*	*q*_1_	*q*_2_
Archid	0.95	0.62	0.3942	0.9971	0.9997
Xi Bootis	0.9	0.66	0.49	0.9988	0.9998
Kruger 60	0.271	0.176	0.3937	0.99992	0.99996

Next, we now compute the positions of the triangular equilibrium points for the binaries **Archid**, **Xi Bootis** and **Kruger 60** in Table 2, 3 and 4, respectively when $r = \sqrt {1 - \mu + \mu^{2} }$, *T* = 0.01 and for the chosen model parameters.

**Table 2:** Coordinates of triangular equilibrium points for **Archid.**

Binary system	Mass ratio *µ*	*k*_1_	*k*_2_	*k*_3_	*q*_1_	*q*_2_	*ε*′	*M*_*b*_	*x*	*y*
Arc hid	0.3942	1.58302 × 10^−7^	9.83933 × 10^−18^	3.1315 × 10^−8^	0.9971	0.9997	0.0001	0.00	− 0.1067	0.4817
	0.3942	1.58302 × 10^−7^	9.83933 × 10^−18^	3.1315 × 10^−8^	0.9971	0.9997	0.0001	0.02	− 0.1067	0.4731
	0.3942	1.58302 × 10^−7^	9.83933 × 10^−18^	3.1315 × 10^−8^	0.9971	0.9997	0.0001	0.04	− 0.1067	0.4644
	0.3942	1.58302 × 10^−7^	9.83933 × 10^−18^	3.1315 × 10^−8^	0.9971	0.9997	0.0001	0.06	− 0.1067	0.4558
	0.3942	1.58302 × 10^−7^	9.83933 × 10^−18^	3.1315 × 10^−8^	0.9971	0.9997	0.0001	0.08	− 0.1067	0.4472
	0.3942	1.58302 × 10^−7^	9.83933 × 10^−18^	3.1315 × 10^−8^	0.9971	0.9997	0.0001	0.10	− 0.1067	0.4385
	0.3942	0.00	0.00	0.00	1	1	0.0001	0.00	0.1058	0.4811
	0.3942	1.58302 × 10^−7^	9.83933 × 10^−18^	3.1315 × 10^−8^	0.9971	0.9997	0.01	0.02	− 0.1067	0.4693
	0.3942	1.58302 × 10^−7^	9.83933 × 10^−18^	3.1315 × 10^−8^	0.9971	0.9997	0.01	0.04	− 0.1067	0.4606
	0.3942	1.58302 × 10^−7^	9.83933 × 10^−18^	3.1315 × 10^−8^	0.9971	0.9997	0.05	0.06	− 0.1067	0.4366
	0.3942	1.58302 × 10^−7^	9.83933 × 10^−18^	3.1315 × 10^−8^	0.9971	0.9997	0.05	0.08	− 0.1067	0.4278
	0.3942	1.58302 × 10^−7^	9.83933 × 10^−18^	3.1315 × 10^−8^	0.9971	0.9997	0.1	0.10	− 0.1067	0.4001
	0.3942	1.58302 × 10^−7^	9.83933 × 10^−18^	3.1315 × 10^−8^	0.9971	0.9997	0.1	0.00	− 0.1067	0.4433

**Table 3:** Coordinates of triangular equilibrium points for **Xi Bootis**.

Binary system	Mass ratio *µ*	*k*_1_	*k*_2_	*k*_3_	*q*_1_	*q*_2_	*ε*′	*M*_*b*_	*x*	*y*
Xi Bootis	0.49	1.58302 × 10^−7^	9.83933 × 10^−18^	3.1315 × 10^−8^	0.9988	0.998	0.0001	0.00	− 0.0097	0.4817
	0.49	1.58302 × 10^−7^	9.83933 × 10^−18^	3.1315 × 10^−8^	0.9988	0.998	0.0001	0.02	− 0.0097	0.4730
	0.49	1.58302 × 10^−7^	9.83933 × 10^−18^	3.1315 × 10^−8^	0.9988	0.998	0.0001	0.04	− 0.0097	0.4644
	0.49	1.58302 × 10^−7^	9.83933 × 10^−18^	3.1315 × 10^−8^	0.9988	0.998	0.0001	0.06	− 0.0097	0.4557
	0.49	1.58302 × 10^−7^	9.83933 × 10^−18^	3.1315 × 10^−8^	0.9988	0.998	0.0001	0.08	− 0.0097	0.4470
	0.49	1.58302 × 10^−7^	9.83933 × 10^−18^	3.1315 × 10^−8^	0.9988	0.998	0.0001	0.10	− 0.0097	0.4383
	0.49	0.00	0.00	0.00	1	1	0.0001	0.00	− 0.0100	0.4811
	0.49	1.58302 × 10^−7^	9.83933 × 10^−18^	3.1315 × 10^−8^	0.9988	0.998	0.01	0.02	− 0.0097	0.4692
	0.49	1.58302 × 10^−7^	9.83933 × 10^−18^	3.1315 × 10^−8^	0.9988	0.998	0.01	0.04	− 0.0097	0.4605
	0.49	1.58302 × 10^−7^	9.83933 × 10^−18^	3.1315 × 10^−8^	0.9988	0.998	0.5	0.06	− 0.0097	0.2633
	0.49	1.58302 × 10^−7^	9.83933 × 10^−18^	3.1315 × 10^−8^	0.9988	0.998	0.5	0.08	− 0.0097	0.2546
	0.49	1.58302 × 10^−7^	9.83933 × 10^−18^	3.1315 × 10^−8^	0.9988	0.998	0.1	0.10	− 0.0097	0.3999
	0.49	1.58302 × 10^−7^	9.83933 × 10^−18^	3.1315 × 10^−8^	0.9988	0.998	0.1	0.00	− 0.0097	0.4433

**Table 4:** Coordinates of triangular equilibrium points for **Kruger 60**.

Binary system	Mass ratio *µ*	*k*_1_	*k*_2_	*k*_3_	*q*_1_	*q*_2_	*ε*′	*M*_*b*_	*x*	*y*
Kruger 60	0.3937	1.58302 × 10^−7^	9.83933 × 10^−18^	3.1315 × 10^−8^	0.99992	0.99997	0.0001	0.00	− 0.1058	0.4811
	0.3937	1.58302 × 10^−7^	9.83933 × 10^−18^	3.1315 × 10^−8^	0.99992	0.99997	0.0001	0.02	− 0.1058	0.4725
	0.3937	1.58302 × 10^−7^	9.83933 × 10^−18^	3.1315 × 10^−8^	0.99992	0.99997	0.0001	0.04	− 0.1058	0.4639
	0.3937	1.58302 × 10^−7^	9.83933 × 10^−18^	3.1315 × 10^−8^	0.99992	0.99997	0.0001	0.06	− 0.1058	0.4552
	0.3937	1.58302 × 10^−7^	9.83933 × 10^−18^	3.1315 × 10^−8^	0.99992	0.99997	0.0001	0.08	− 0.1058	0.4466
	0.3937	1.58302 × 10^−7^	9.83933 × 10^−18^	3.1315 × 10^−8^	0.99992	0.99997	0.0001	0.10	− 0.1058	0.4380
	0.3937	0.00	0.00	0.00	1	1	0.0001	0.00	− 0.1058	0.4807
	0.3937	1.58302 × 10^−7^	1.58302 × 10^−7^	3.1315 × 10^−8^	0.99992	0.99997	0.01	0.02	− 0.1058	0.4687
	0.3937	1.58302 × 10^−7^	1.58302 × 10^−7^	3.1315 × 10^−8^	0.99992	0.99997	0.01	0.04	− 0.1058	0.4600
	0.3937	1.58302 × 10^−7^	1.58302 × 10^−7^	3.1315 × 10^−8^	0.99992	0.99997	0.5	0.06	− 0.1058	0.2628
	0.3937	1.58302 × 10^−7^	1.58302 × 10^−7^	3.1315 × 10^−8^	0.99992	0.99997	0.5	0.08	− 0.1058	0.2542
	0.3937	1.58302 × 10^−7^	1.58302 × 10^−7^	3.1315 × 10^−8^	0.99992	0.99997	0.1	0.10	− 0.1058	0.3995
	0.3937	1.58302 × 10^−7^	1.58302 × 10^−7^	3.1315 × 10^−8^	0.99992	0.99997	0.1	0.00	− 0.1058	0.4427

**Figure 2:**
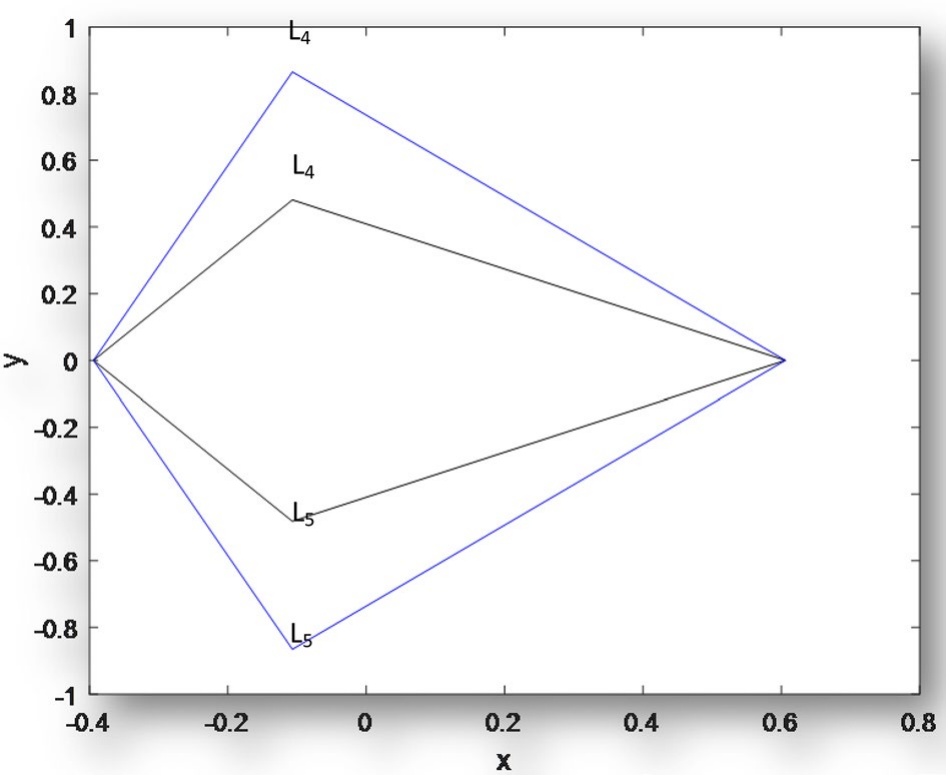
Coordinates of Triangular points for Archid when $\mu = 0.3942,q_{1} = q_{2} = 1,k_{i} (i = 1,2,3 = 0.00),\varepsilon ^{\prime } = 0.00,M_{b} = 0.00$ and the inner when $\mu = 0.3942,M_{b} = 0.00$, $q_{1} = 0.9971,{\text{ }}q_{2} = 0.9997$, $\varepsilon ^{\prime } = 0.0001$, $k_{1} = 1.58302 \times 10^{{ - 7}} , k_{2} = 9.83933 \times 10^{{ - 18}}, k_{3} = 3.13153 \times 10^{{ - 8}}$.

**Figure 3:**
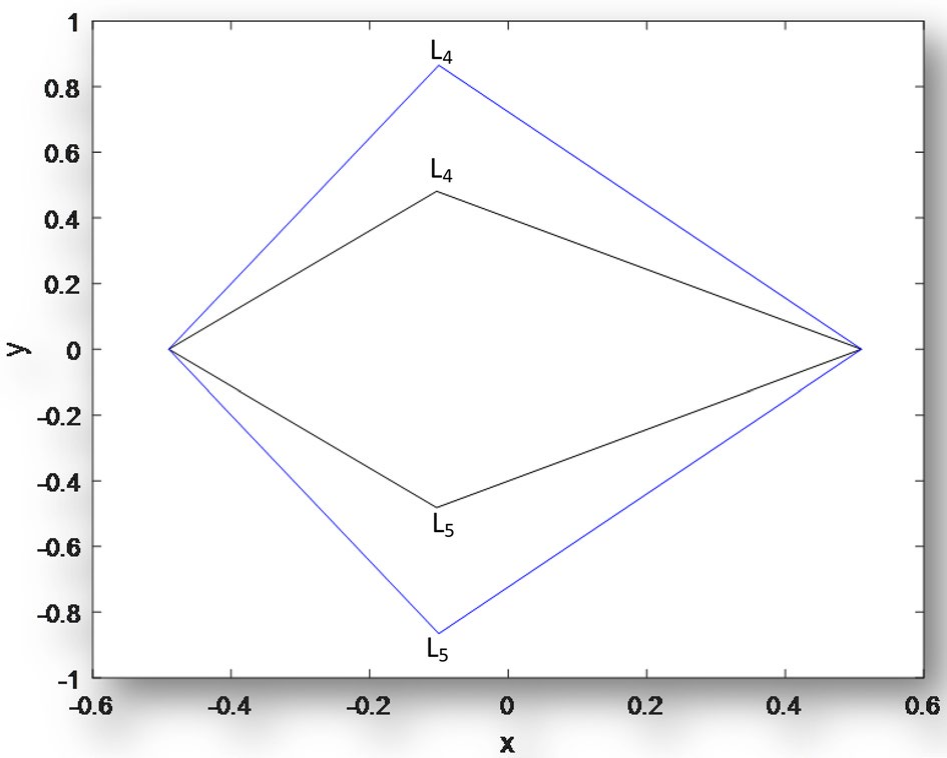
Coordinates of Triangular points for **Xi Bootis** when $\mu = 0.49,{\text{ }}q_{1} = q_{2} = 1,{\text{ }}k_{i} (i = 1,2,3 = 0.00), \varepsilon ^{\prime } = 0.00,M_{b} = 0.00$ and the inner when $\mu = 0.49,M_{b} = 0.00, q_{1} = 0.9988,q_{2} = 0.9988, \varepsilon ^{\prime } = 0.0001, k_{1} = 1.58302 \times 10^{{ - 7}}, k_{2} = 9.83933 \times 10^{{ - 18}}, k_{3} = 3.13153 \times 10^{{ - 8}}.$

**Figure 4:**
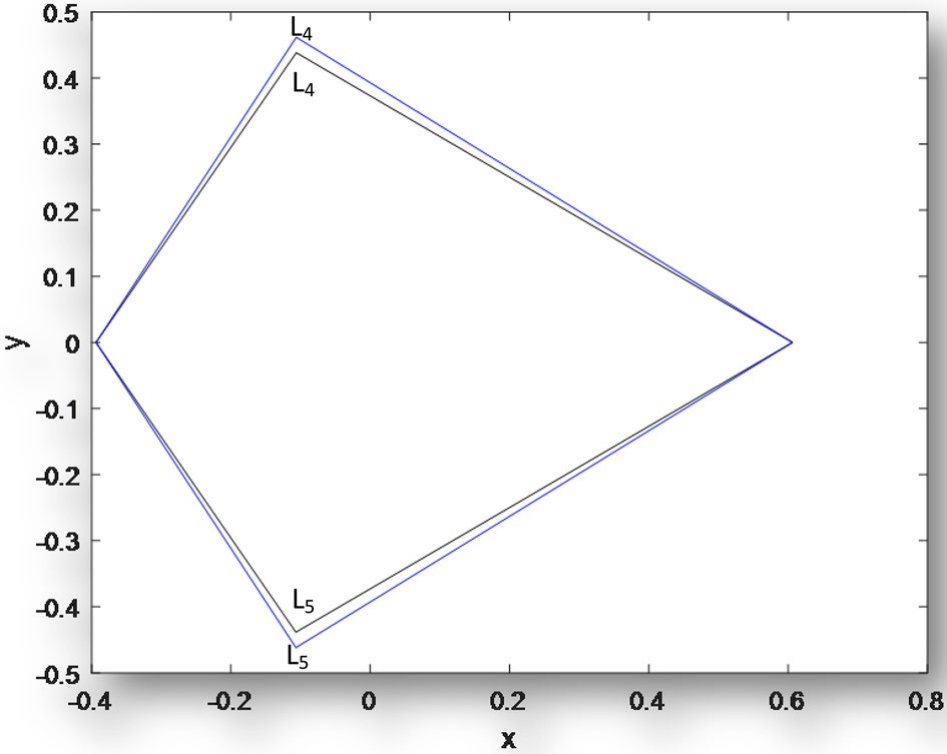
Coordinates of Triangular points for **Kruger 60** when $\mu = 0.3937,q_{1} = q_{2} = 1, k_{i} (i = 1,2,3 = 0.00),\varepsilon ^{\prime } = 0.00,M_{b} = 0.00$ and the inner figure shows when $\mu = 0.3937,M_{b} = 0.00$, *q*_1_ = 0.9999, *q*_2_ = 0.9999, $\varepsilon ^{\prime } = 0.0001, k_{1} = 1.58302 \times 10^{{ - 7}}, k_{2} = 9.83933 \times 10^{{ - 18}}, k_{3} = 3.13153 \times 10^{{ - 8}}$.

Next, we plot the coordinates of the triangular equilibrium points for the binaries Archid,

Xi-Bootis and Kruger 60 in Figs. 2, 3 and 4, respectively. These figures have been drawn using the numerical computations in Tables 2, 3 and 4, respectively.

Figure 2 is a plot of the coordinates of triangular points for **Archid**. The outer curve represents the triangular equilibruim points of the classical R3BP when there are no heterogenous layers and the primaries are non emitters of radiation pressure while the inner curve shows the combined effects of the model paramters.Same results are observed in Figs. 3 and 4 for **Xi Bootis** and **Kruger 60.**

## Linear stability of triangular points

The motion of an infinitesimal particle near any equilibrium point is said to be stable if given a small displacement with a small velocity, the particle oscillates considerably about the point and stays around for all time, else it is said to be unstable. The motion of the infinitesimal particle in the *xy*-plane is investigated by giving the triangular point $(x_{0,} y_{0} )$, small displacements, $\xi$ , $\zeta$ in the coordinates, Then we can write

13 $$x = x_{0} + \xi \quad y = y_{0} + \zeta$$

Now substituting these values in Eq. (1) and expanding its right hand side, we obtain the variational equations of motion as,

14 $$\begin{gathered} \ddot{\xi } - 2n\alpha \dot{\zeta } = (\Omega_{xx}^{0} )\xi + (\Omega_{xy}^{0} )\zeta \hfill \\ \ddot{\zeta } + 2n\alpha \dot{\xi } = (\Omega_{yx}^{0} )\xi + (\Omega_{yy}^{0} )\zeta \hfill \\ \end{gathered}$$

Only linear terms in $\xi$ and $\zeta$ have been considered. The second partial derivatives of Ω are denoted by subscripts. The superscript 0 indicates that the derivatives are evaluated at the triangular point $(x_{0,} y_{0} )$ .

The characteristic equation corresponding to Eq. (14) can be written as

15 $$\lambda^{4} - \left( {\Omega_{xx}^{0} + \Omega_{yy}^{0} - 4n^{2} \alpha^{2} } \right)\lambda^{2} + \Omega_{xx}^{0} \Omega_{yy}^{0} - \left( {\Omega_{xy}^{0} } \right)^{2} = 0$$

The values of partial derivatives at the triangular points (12) are:

$$\Omega_{xx}^{0} = \frac{3}{4} + \frac{3}{4}a_{1} + \frac{3}{4}\mu b_{1} + \frac{15}{8}\left( {k_{1} + k_{2} } \right) + \frac{{3M_{b} (r_{c}^{2} - \frac{3}{4})}}{{\left( {r_{c}^{2} + T^{2} } \right)^{\frac{5}{2}} }}$$

$$\Omega_{yy}^{0} = \frac{9}{4} + \frac{9}{4}a_{2} + \frac{1}{4}\mu b_{2} + \frac{33}{8}\left( {k_{1} + k_{2} } \right) + \frac{{3k_{3} }}{2} + \frac{{M_{b} \left( {2r_{c} - 1} \right)}}{{\left( {r_{c}^{2} + T^{2} } \right)^{\frac{3}{2}} }} + \frac{{3M_{b} (\frac{3}{4})}}{{\left( {r_{c}^{2} + T^{2} } \right)^{\frac{5}{2}} }}$$

$$\Omega_{xy}^{0} = \frac{ - 3\sqrt 3 }{4}\left[ {a_{3} + \mu b_{3} + \frac{{5(k_{1} - k_{2} )}}{2} - \frac{{M_{b} (1 - 2\mu )}}{{\left( {r_{c}^{2} + T^{2} } \right)^{\frac{5}{2}} }}} \right]$$

where $$a_{1} = \frac{5}{3}\varepsilon^{\prime} - p_{1} - \varepsilon_{1} - 4\varepsilon_{2}$$

$$b_{1} = p_{1} - p_{2} - 3\varepsilon_{1} + 3\varepsilon_{2}$$

$$a_{2} = \frac{7}{9}\varepsilon^{\prime } + \frac{{4\varepsilon_{2} }}{3} - \frac{{5p_{1} }}{9} - \frac{{21\varepsilon_{1} }}{9}$$

$$b_{2} = 5p_{1} - 5p_{2} - 33\varepsilon_{2} + 33\varepsilon_{1}$$

$$a_{3} = 1 - p_{1} + \frac{{11\varepsilon^{\prime}}}{9} - \frac{{7\varepsilon_{1} }}{3} - \frac{{4\varepsilon_{2} }}{3},$$

$$b_{3} = 2 - p_{1} - p_{2} + \frac{{22\varepsilon^{\prime}}}{9} - \frac{{11\varepsilon_{1} }}{3} - \frac{{11\varepsilon_{2} }}{3}$$

Here each of $\left| {a_{j} } \right|$, $\left| {b_{j} } \right|$ $(j = 1,2,3)$ is very small , as $\left| {\varepsilon_{i} } \right| < < 1$, $\left| {\varepsilon^{\prime}} \right| < < 1$ and $\left| {p_{i} } \right| < < 1$, $(i = 1,2)$

Substituting these values in Eq. (15), then the characteristic equation becomes,

16 $$\lambda^{4} + h_{1} \lambda^{2} + h_{2} = 0$$

where

$$h_{1} = 4n^{2} \alpha^{2} - \left( {\Omega_{xx}^{0} + \Omega_{yy}^{0} } \right) = 1 + 8\varepsilon - 3\varepsilon^{\prime} - 3\left( {k_{1} + k_{2} } \right) + \frac{{3k_{3} }}{2} + \frac{{M_{b} \left( {2r_{c} + 3} \right)}}{{\left( {r_{c}^{2} + T^{2} } \right)^{\frac{3}{2}} }} - \frac{{M_{b} 3r_{c}^{2} }}{{\left( {r_{c}^{2} + T^{2} } \right)^{\frac{5}{2}} }}$$

$$\begin{aligned} & h_{2} = \Omega_{xx}^{0} \Omega_{yy}^{0} - \left( {\Omega_{xy}^{0} } \right)^{2} = \frac{27\mu }{4} - \frac{{27\mu^{2} }}{4} + \frac{{33\mu \varepsilon^{^{\prime}} }}{2} - \frac{{33\mu^{2} \varepsilon^{^{\prime}} }}{2} + \frac{3\mu }{2}\left( {p_{1} + p_{2} } \right) - \frac{{3\mu^{2} }}{2}\left( {p_{1} + p_{2} } \right) + \frac{9\mu }{2}\left( {k_{1} - k_{2} } \right) \\ & \quad + \frac{{9k_{2} }}{2} + \frac{{99k_{3} }}{4}\left( {\mu - \mu^{2} } \right) + \frac{{33\mu \left( {1 - \mu } \right)\left( {2r_{c}^{2} - 1} \right)M_{b} }}{{2\left( {r_{c}^{2} + T^{2} } \right)^{\frac{3}{2}} }} + \frac{{27\mu \left( {1 - \mu } \right)M_{b} }}{{4\left( {r_{c}^{2} + T^{2} } \right)^{\frac{5}{2}} }} \\ \end{aligned}$$

Its roots are

17 $$\lambda^{2} = \frac{{ - h_{1} \pm \sqrt \Delta }}{2}$$

where

18 $$\Delta = h_{1}^{2} - 4h_{2}$$

$$\begin{aligned} & \Delta = \left[ {27 + 66\varepsilon^{\prime } + 6p_{1} + 6p_{2} + 99k_{3} + \frac{{66M_{b} \left( {2r_{c} - 1} \right)}}{{\left( {r_{c}^{2} + T^{2} } \right)^{\frac{3}{2}} }} + \frac{{27M_{b} }}{{\left( {r_{c}^{2} + T^{2} } \right)^{\frac{5}{2}} }}} \right]\mu^{2} \\ & \quad - \left( {27 + 66\varepsilon^{\prime } + 6p_{1} + 6p_{2} + 18k_{1} - 18k_{2} + 99k_{3} + \frac{{66M_{b} \left( {2r_{c} - 1} \right)}}{{\left( {r_{c}^{2} + T^{2} } \right)^{\frac{3}{2}} }} + \frac{{108M_{b} }}{{\left( {r_{c}^{2} + T^{2} } \right)^{\frac{5}{2}} }}} \right)\mu \\ & \quad + 1 + 16\varepsilon - 6\varepsilon^{\prime } - 6\left( {k_{1} + k_{2} } \right) + 3k_{3} - 18k_{2} + \frac{{2M_{b} \left( {2r_{c} + 3} \right)}}{{\left( {r_{c}^{2} + T^{2} } \right)^{\frac{3}{2}} }} - \frac{{6M_{b} r_{c}^{2} }}{{\left( {r_{c}^{2} + T^{2} } \right)^{\frac{5}{2}} }} \\ \end{aligned}$$

Now $\Delta$ is a strictly decreasing function of $\mu$ in the interval $\left( {0,\frac{1}{2}} \right)$ and it has values of opposite signs at the end points, hence there is only one value of $\mu$ , say $\mu_{C}$ in the interval $\left( {0,\frac{1}{2}} \right)$ for which the discriminant vanishes. $\mu_{C}$ is called the critical mass parameter and it is given by

20 $$\mu_{C} = \mu_{0} + \mu_{h} + \mu_{r} + \mu_{p} + \mu_{b}$$

with

$$\mu_{0} = \frac{1}{2}\left( {1 - \frac{{\sqrt {23} }}{{\sqrt {27} }}} \right)$$

$$\mu_{h} = - \frac{1}{3}\left[ {\left( {1 + \frac{15}{{\sqrt {69} }}} \right)k_{2} - \left( {1 - \frac{15}{{\sqrt {69} }}} \right)k_{1} } \right] - \frac{2}{{9\sqrt {69} }}k_{3}$$

$$\mu_{r} = - \frac{{2\left( {p_{1} + p_{2} } \right)}}{{27\sqrt {69} }}$$

$$\mu_{p} = \frac{{4(36\varepsilon - 19\varepsilon^{\prime})}}{{27\sqrt {69} }}$$

$$\mu_{b} = \frac{{\left[ {4\left( {19 - 2r_{c} } \right)\left( {r_{c}^{2} + T^{2} } \right)^{\frac{5}{2}} - 9\left( {1 + 6r_{c}^{2} } \right)\left( {r_{c}^{2} + T^{2} } \right)^{\frac{3}{2}} } \right]M_{b} }}{{27\sqrt {69} \left( {r_{c}^{2} + T^{2} } \right)^{4} }}$$

The first term $\mu_{0}$ represents Routh’s critical mass value; the second term $\mu_{h}$ is the effect arising from heterogeneity of both primaries, the third term $\mu_{r}$ denotes the effect of the radiation pressure of both primaries; the fourth term $\mu_{p}$ is the measure of the impact of small perturbations in Coriolis and centrifugal forces; the last term is due to the gravitational potential from the circumbinary disc (belt).

Now if $$h_{1} > 0,$$$$\Delta > 0$$ in the interval $$0 < \mu < \mu_{C}$$ the roots (17) are distinct imaginary numbers. Hence the triangular points are stable in this region.

If $\mu_{C} < \mu < \frac{1}{2}$, $\Delta < 0,$ then the real parts of two of the roots (17) are positive. Therefore, the triangular points are unstable. If $\mu = \mu _{C}$, $\Delta = 0$, then the roots (17) are double roots, which gives instability of the points.

Hence we have established that the triangular points are stable only when the mass ratio $\mu \in \left( {0,\mu _{C} } \right)$ and unstable when the mass ratio $\mu \in \left[ {\mu _{C} ,\frac{1}{2}} \right]$, where $\mu _{C}$ is the critical mass ratio which depends on the combined effect of perturbations in the Coriolis and centrifugal forces, heterogeneity and radiation pressure of the primaries and gravitational potential from a belt.

Using the software *Mathematica*, we compute the effects of the various perturbations on the critical mass value, when $T = 0.01$, $r_{c} = 0.99$ (Table 5).

**Table 5:** Numerical computations of the critical mass *µ*_*C*_ under different characterizations.

Case	*q*_1_	*q*_2_	*k*_1_	*k*_2_	*k*_3_	*ε*	*ε*′	*M*_*b*_	*µ*_*C*_
1	1	1	0	0	0	0	0	0	0.038520
2	1	1	1.58302 × 10^−7^	9.83933 × 10^−8^	3.1315 × 10^−8^	0.01	0.01	0.01	0.0212269
3	1	1	0	0	0	0	0	0	0.020686
4	0.98	1	0	0	0	0	0	0	0.0208643
5	0.93	1	0	0	0	0	0	0	0.0213102
6	0.88	1	0	0	0	0	0	0	0.0217561
7	1	0.95	0	0	0	0	0	0	0.0211318
8	1	0.90	0	0	0	0	0	0	0.0215777
9	1	0.85	0		0	0	0	0	0.0220236
10	1	1	1.58302 × 10^–7^	0	0	0	0	0	0.0209891
11	1	1	0	9.83933 × 10^–8^	0	0	0	0	0.0206859
12	1	1	0	0	3.1315 × 10^–8^	0	0	0	0.0206861
13	1	1	0	0	0	0.01	0	0	0.0215533
14	1	1	0	0	0	0	0.01	0	0.0205724
15	1	1	0	0	0	0	0	0.01	0.0209113

## Discussion

Equation (1) describes motion of a third body under combined effects of radiation pressures and heterogeneity of the primaries, small perturbations and potential from a massive belt. Equation (12) gives positions of triangular equilibrium points which are defined by the radiation pressures, heterogeneity of primaries, a small deflection in the centrifugal force and the potential from a belt. These points are not affected by a small perturbation in the Coriolis force because the Eq. (12) is independent of the parameter *α*. It may be noted here that in this problem, the triangular points do not form equilateral triangles with the primaries as they do in the classical case, rather they form scalene triangles with the primaries.

Equation (20) gives the value of the critical mass parameter, $\mu _{c}$ of the system and depends on small perturbations in the Coriolis and centrifugal forces, heterogeneity of the primaries, radiation factor and potential from a belt. The critical mass parameter is used to determine the region of the stability of the triangular points and also helps in analysing the behaviour of the parameters involved. Hence, the triangular points are stable for 0 < *μ*< $\mu _{c}$ and when this happens, the Coriolis force and the belt have stabilising tendencies, while the centrifugal force, radiation pressure and heterogeneity of the primaries have destabilisingbehaviours. However, the overall effect is that the region of stable motion decreases in the presence of these destabilizing perturbations.

The expressions for $\mu _{{c1}}$ and $\mu _{{c2}}$ in Suraj et al.^12^ are presented in the corrected form in Eq. (20). In the case when the heterogeneity of the primaries is ignored, the results presented here are in agreement with those of Singh and Taura^11^, if its oblateness are absent. In the absence of both heterogeneity of the primaries and the potential from a belt. Also, our results are in agreement with those of Singh and Haruna^14^, if its bodies are spherical. In the absence of drag force, perturbations and heterogeneity of the primaries, while the primaries are luminous and there is effect of potential from a belt, our results coincide with those obtained by Singh and Amuda^3^.

## Conclusion

The paper investigates positions and linear stability of triangular points when both primaries are heterogeneous and luminous and surrounded by a belt under effects of small perturbations in the Coriolis and centrifugal forces. It is seen that the positions are not affected by a small perturbation in the Coriolis force. Motion around triangular equilibrium points is stable only when $0 < \mu < \mu _{c}$, where $\mu _{c}$ is defined by the radiation pressures, heterogeneity of the primaries, small perturbations, and potential from a belt. We observed that the Coriolis force and the belt possess stabilizing tendencies, while the centrifugal force, radiation and heterogeneity of the primaries are destabilising. A practical application of this model is motion of a dust grain near a heterogeneous and luminous binary stars surrounded by a belt.
